# Genetic analysis of keel bone fractures in laying hens housed in a quasi-commercial aviary

**DOI:** 10.1016/j.psj.2025.106067

**Published:** 2025-11-07

**Authors:** Pascal Duenk, Henk Bovenhuis, Pauline Willemsen, Bayode O. Makanjuola, Matthew B. Petelle, Sabine G. Gebhardt-Henrich, Christine F. Baes, Michael J. Toscano

**Affiliations:** a Animal Breeding and Genomics, Wageningen University & Research, Droevendaalsesteeg 1 6708PB Wageningen, the Netherlands; b Institut de Selection Animale B.V., Spoorstraat 69 5831CK Boxmeer, the Netherlands; c Centre for Genetic Improvement of Livestock, Department of Animal Biosciences, University of Guelph, 50 Stone Road East N1G 2W1 Guelph, Canada; d Center for Proper Housing: Poultry and Rabbits (ZTHZ), Division of Animal Welfare, VPH Institute, University of Bern, Burgerweg 22,3052 Zollikofen, Switzerland; e Institute of Genetics, Department of Clinical Research and Veterinary Public Health (DCR-VPH), University of Bern, Bremgartenstrasse 109A 3012 Bern, Switzerland

**Keywords:** Bone health, Genetics, Genome-wide association, Radiograph, Maturity

## Abstract

Keel bone fractures (KBF) in laying hens are a major welfare problem in the egg production industry. While housing design and nutrition are known contributing factors, the role of genetics in KBF susceptibility is not well understood. The objective of this study was to estimate the heritability of KBF susceptibility and identify associated genomic regions and candidate genes in commercial laying hens. We used KBF assessment scores derived from radiographs of 1,060 white-feathered two-way crossbred hens housed in a quasi-commercial aviary system. A log-transformation was applied to the KBF scores to normalize the data. All hens were genotyped with a 60 K SNP chip. We fit a linear mixed model with a genomic relationship matrix to estimate variance components and heritability, and conducted a genome-wide association study to identify SNPs associated with KBF. Our results showed that KBF was low to moderately heritable, with an estimated heritability of 0.08 on the original trait scale, and 0.22 on the log-transformed scale. We identified four significant or suggestive haplotype blocks on chromosomes 2 and 20 that together explained 13.2 % of the total additive genetic variance. These blocks contain several candidate genes (including BCAS1, CYP24A1, PFND4, TSHZ2, and GDF6) that have been linked to calcium and vitamin D homeostasis, skeletal development, and bone density in humans and mice. These findings support the possibility that susceptibility to KBF is determined not only by genes influencing bone strength directly, but also by genes affecting the vitamin D metabolic pathway that regulates calcium reabsorption. Finally, one of the genomic regions on chromosome 20 was in close proximity to QTL that have previously been associated with egg production levels and age at sexual maturity in chickens. Overall, our findings contribute to our understanding of the genetic architecture of KBF susceptibility, its possible relationship to early egg production, and the physiological pathways that influence skeletal health in laying hens.

## Introduction

Keel bone fractures (KBF) are a widespread phenomenon in laying hens. The extremely high prevalences of up to 97 % in non-cage systems in combination with the associated discomfort makes KBF one of the greatest welfare concerns in the egg production industry (EFSA et al., 2023; Toscano et al., 2020). KBF occur more often in furnished cages and aviaries compared to conventional cages (Hardin et al., 2019; Rufener and Makagon, 2020; Thøfner et al., 2021), and it has therefore been argued that the direct cause for KBF are collisions with housing features such as elevated perches (Baker et al., 2024; Stratmann et al., 2015). However, recent pathological examinations of fractures (Thøfner et al., 2020) and the common occurrence of KBF across different housing systems (Rufener and Makagon, 2020; Thøfner et al., 2021) suggest factors other than collisions play an important role as well (Toscano et al., 2020).

One likely cause for KBF in laying hens is the high calcium demands required for egg shell production (Eusemann et al., 2020). Calcium can be reabsorbed from the bones (Dacke et al., 1993), which compromises the bone integrity and reduces bone health (Jansen et al., 2020b; Zhao et al., 2020). Although this process is not the direct cause of fractures, it does make the bones more susceptible to breakage. However, more recent studies have provided evidence that sustained, high egg production is not the only determining factor contributing to increased susceptibility to fractures. Several studies have shown that the rate of KBF occurrence tends to level off or decrease after the hens reach the age of 49 weeks (Gretarsson et al., 2024; Rufener and Makagon, 2020; Toscano et al., 2020), which represents a period in which hens still produce eggs at a rate above 90 % of peak production. Instead of high levels of egg production, the early onset of lay in contemporary laying hens may be an important factor contributing to increased risk of KBF (Dunn, 2025; Gebhardt-Henrich and Fröhlich, 2015). Laying hens typically start egg production around 18 weeks of age, while the keel bone does not fully ossify until about 32-40 weeks of age (Buckner et al., 1948; Gretarsson et al., 2024). As a result, the calcium required at the start of egg production may prevent or delay full ossification of the keel bone leading to a weakened structure and increasing susceptibility to fractures (Gebhardt-Henrich and Fröhlich, 2015).

Susceptibility to KBF varies across genetic lines and individuals (Candelotto et al., 2017). For example, KBF prevalence is on average about 1.5 times higher in commercial brown-feathered hens compared to white-feather laying hens (Rufener and Makagon, 2020). This difference in KBF susceptibility has been attributed to between-line differences in keel morphology and bone density (Rufener and Makagon, 2020; Thøfner et al., 2021). Moreover, significant within-line variability in fracture occurrence reflects the presence of variation in KBF susceptibility at the individual level (Thøfner et al., 2021). Finally, genetic analyses in chickens have demonstrated moderate to high heritabilities for bone strength and density traits, ranging from 0.13 to 0.58 (Jansen et al., 2020a; Johnsson, 2020), and have uncovered QTLs and candidate genes involved in mineralization, collagen cross-linking, and osteoblast/osteoclast regulation (Johnsson et al., 2015; Raymond et al., 2018). Together, these observations suggest that variation in KBF susceptibility between individuals of the same line has an important genetic component.

Understanding the genetics of KBF susceptibility can reveal its biological causes and inform prevention strategies. However, dedicated studies on the genetic architecture of KBF remain scarce. There are no published estimates of heritability of KBF susceptibility, and no genome-wide association studies for this trait. Consequently, potential candidate genes and pathways driving KBF susceptibility are still uncharacterized. Moreover, assessment methods are diverse with variable levels of accuracy likely leading to poor characterization across and within studies (Rufener and Makagon, 2020). The objective of the present study was to quantify genetic variance components and heritability of KBF susceptibility and to identify genomic regions and candidate genes influencing this trait. Our study used radiographic images which allow for clear examination of the bone in live hens that can be shared across multiple groups for validation (or automation) leading to a higher quality phenotype (Casey-Trott et al., 2015). We analyzed phenotype and genotype data from 1,060 white-feathered hens housed in a quasi- commercial aviary system.

## Materials and methods

### Animals

We used data from a large research project led by the University of Bern that collects tracking and health assessment data of a large number of laying hens housed in a quasi-commercial aviary (Open Philantropy, 2025). The chickens were provided by Hendrix Genetics (Boxmeer, The Netherlands) and included 4,800 white two-way crossbred laying hens. These hens were offspring of 100 sires from a purebred parental line, which were each randomly mated to 16 dams from a different purebred parental line. Both the sire and dam line are used in the commercial breeding program to produce Dekalb White hens. The hens were hatched in June 2021 and arrived as hatchlings at the Aviforum site in Switzerland, after which they were placed into eight pens in an on-site rearing barn, stratified by sire. Four of the rearing pens had the Landmeco Harmony rearing aviary (Landmeco A/S, Olgod, Denmark, 4.89 × 4.55 m), and the other four had the Inauen Natura rearing aviary (R. Inauen AG, Appenzell, Switzerland, 4.86 × 3.92 m). After approximately 11 weeks, the birds were offered daytime access to a winter garden with perches. At approximately 18 weeks of age, all hens were transferred to a production barn divided into twenty equally sized pens. However, only hens of five pens were used in this study, each housing approximately 225 hens. These hens were offspring from 25 sires that represented the extremes (high and low) of a predefined index value, which included breeding values for livability and survivability. This selection procedure was adopted to maximize variation in hen quality, which was necessary for another study on different traits using the same population. Each of the five pens served as a replicate, stratified by sire and the original rearing barn pen. The production barn featured a Bolegg Terrace aviary (Vencomatic Group, 5521 DW Eersel, The Netherlands), which included a top-level tier, nest box tier, lower-level tier, a floor-littered area, and an attached winter garden area. The aviary was further adapted to Swiss regulations by moving the drinking line normally positioned in front of the nestbox to the top-level tier. Feeding, vaccination, light duration, and other management procedures followed standard guidelines for the Dekalb White hybrid. The maximum light duration was 14 hours, from 03:00 to 17:00, with natural light supplemented by artificial light. Access to the winter garden was provided between 10:00 and 16:00.

### Phenotype data

All hens that were still alive at 30 weeks of age (*N* = 1,073) were caught, crated, assessed for feather damage and footpad health, and individually radiographed. Radiographs were collected using a portable X-ray machine (Gierth X-Ray international GmbH) set to 54 kV and 2.8mAs, and with a focus distance of 80-82 cm. Each hen was hung upside down in a metal shackle suspended from a wooden frame which induced immobility (Baur et al., 2020; Rufener et al., 2018). After taking the radiographs, hens were immediately removed from the shackle, crated, and placed back into their original pen. Radiographs were exported as DICOMs and each latero-lateral x-ray was scored by a single rater who had been trained to score keels using an established protocol (Rufener et al., 2018). A trained rater scored each radiograph for the aggregate severity of all present fractures. The scoring system was based on a tagged visual analogue scale (tVAS) with a continuous range from 0.0 (healthy, free from fractures) to 10.0 (severe and many fractures). The rater used a catalog of example images to help determine the score, which represented the total amount of bone affected by fractures.

From the total of 1073 records, we removed 11 records that either had a missing value for pen or rearing pen, and 2 records that did not have a matching genotype record. In the remaining 1060 records, the date of recording (5 days within a week) was almost entirely confounded with pen, except for one observation. The distribution of KBF score was strongly right-skewed, so we decided to transform the data. Several transformations (log, Box-Cox, Yeo-Johnsson) resulted in similar distributions, so we decided to use a log transformation for ease of interpretability. The data was transformed using the formula

(1) $$logKBFScore=\log_{e}\left(KBFScore+0.05\right).$$

We added 0.05 (the smallest observed value on the original scale) to the KBF scores to enable log-transformation of zero values. Both KBFScore as well as logKBFScore were used for estimating variance components and heritability, while only the log-transformed scores were used for genome-wide association.

### Genotype data

Hens that were alive at 30 weeks of age were genotyped using a proprietary 60 K SNP panel (Illumina Inc.). The genotype data was subjected to quality control through the following procedure. First, we removed two genotypes of individuals that did not have phenotypes, leaving 1,060 hens with both phenotype and genotype data. Second, SNPs were removed if they had a minor allele frequency lower than 0.02, a call rate below 90 %, or were heterozygous for all individuals. Finally, we removed SNPs that were unmapped, or that were located on the sex chromosomes. After this quality control, 39,493 SNPs remained for further analysis. Note that we did not remove any SNPs based on deviations from Hardy-Weinberg Equilibrium (HWE), because the hens in this study were all two-way crossbreds, so deviations from HWE were expected.

The genotypes were used to construct a genomic relationship matrix (G), using method 1 of VanRaden (2008)

(2) $$G=\frac{{\mathrm{ZZ}}^{\prime }}{\sum 2p_{i}\left(1-p_{i}\right)},$$

where Z is matrix of centered allele counts (coded as 0, 1, or 2) with individuals in rows and SNPs in columns, and $p_{i}$ is the allele frequency of SNP i. To analyze population structure, we performed a principal component analysis (PCA) on the G matrix, and plotted the first two principal components against each other.

### Estimating variance components and heritability

Variance components for KBFScore and logKBFScore were estimated using the model

(3) y=μ+Xb+Zu+e,

where y is a phenotypes (KBFScoreor logKBFScore), μ is the overall mean, b is a vector of fixed effects for pen and rearing pen with incidence matrix X, u is a vector of random additive genetic effects with incidence matrix Z, and e is a vector of random residuals. The distribution of additive genetic effects is assumed $u\;∼\;N(0,\;G\sigma_{u}^{2}$), where G is the genomic relationship matrix, and $\sigma_{u}^{2}$ is the additive genetic variance. The distribution of residuals is assumed $e\;∼\;N(0,\;I\sigma_{e}^{2})$, where I is an identity matrix, and $\sigma_{e}^{2}$ is the residual variance. Variance components were estimated using restricted maximum likelihood (REML) using the software ASReml 4.2.1 (Gilmour et al., 2021).

### Genome-wide association study (GWAS)

We tested each SNP for an association with log*KBFScore* using the genotypic model in PLINK 1.9 (Purcell, 2015). To improve model stability, we corrected for fixed effects by first performing a PCA on the design matrix of fixed effects (pen and rearing pen), and then including the first 11 principle components (explaining more than 99 % of the variation in fixed effects) in the model for testing SNP associations. The model was

(4) $$y=\mu +X_{PC}b_{pc}+z_{i}a_{i}+w_{i}d_{i}+e$$

where μ is the overall mean, $b_{PC}$ is a vector of regression coefficients for the principal components with incidence matrix $X_{PB}$, and e is a vector of random residuals, distributed as $e\;∼N(0,I\sigma_{e}^{2})$. For SNP i, $z_{i}$ is the column vector of centered allele counts (coded as 0, 1, or 2), $a_{i}$ is the additive genetic effect (which is a regression coefficient), $w_{i}$ is the column vector containing 1 for heterozygous individuals and 0 for homozygous individuals, and $d_{i}$ is the dominance effect (which is a regression coefficient). To empirically correct for genomic inflation, we regressed the observed $-\log_{10}\left(p\right)$ values that were smaller than 3 on their theoretically expected $-\log_{10}\left(p\right)$ values, and then divided all observed $-\log_{10}\left(p\right)$ values by the estimated regression coefficient (β) to obtain corrected values. This correction was applied for additive and dominance effects separately.

To correct for multiple testing, we estimated the number of independent SNPs by pruning the data with PLINK 1.9, using a window size of 500 kb, a step size of 10 SNPs, and an R^2^ threshold of 0.2. This pruning process retained 2618 independent SNPs, corresponding to a genome-wide significance threshold of 0.05/2618 = 1.91×10^−5^ and a suggestive threshold of 0.10/2618 = 3.82×10^−5^.

Significant and suggestive SNPs were assigned to haplotype blocks that were calculated based on LD using PLINK 1.9 (Purcell, 2015). The parameters for determining haplotype blocks included a maximum block size of 5000 kb, a lower D’ confidence interval of 0.60, and an upper D’ confidence interval of 0.90. We calculated the proportion of additive genetic variance in logKBFscore explained by SNPs in all significant and suggestive haplotype blocks using the model

(5) $$y=\mu +\mathrm{Xb}+Zu_{1}+Zu_{0}+e,$$

where $u_{1}$ is a vector of random additive genetic effects pertaining to significant and suggestive haplotype blocks, and $u_{0}$ is a vector of random additive genetic effects pertaining to the rest of the genome. We assumed distributions of $u_{1}\;∼\;N(0,\;G_{1}\sigma_{u_{1}}^{2}$) and $u_{0}\;∼\;N\left(0,\;G_{0}\sigma_{u_{0}}^{2}\right)$, where $G_{1}$is a genomic relationship matrix built with all SNPs in significant and suggestive haplotype blocks, and $G_{o}$is a genomic relationship matrix built with all other SNPs. The proportion of additive genetic variance explained by the significant and suggestive haplotype blocks was calculated as $\sigma_{u_{1}}^{2}/\left(\sigma_{u_{1}}^{2}+\sigma_{u_{0}}^{2}\right)$.

We then mapped the haplotype blocks to the chicken genome assembly Galgal6 (GRCg6a), and identified overlapping genes that were less than 50 kb upstream or downstream from the borders of the block. All overlapping genes were used to query the Human GWAS Catalog (version 1.0.2, downloaded from the NHGRI-EBI GWAS Catalog (Cerezo et al., 2024) on 24/03/2025) and the Mouse Genome Informatics (MGI) Database (accessed 07/05/2025 Baldarelli et al., 2024; The Jackson Laboratory, 2025) to identify earlier reported gene-trait associations in humans (*Homo Sapiens)* and mouse (*Mus Musculus)*. In addition, we looked for overlaps with QTL reported in the Chicken QTL database (version 55, downloaded from the Animal QTLdb (Hu et al., 2021) on 10/03/2025).

## Results

### Genomic relationships

**Fig. 1:**
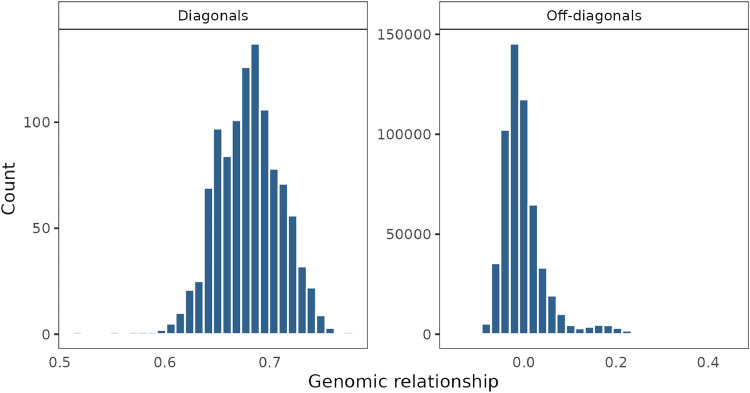
Distribution of genomic relationships. Relationships are separated in diagonals (self-relationships, left panel), and off-diagonals (inter-individual relationships, right panel).

The diagonal elements of the genomic relationship matrix (G) (i.e. self-relationships) ranged from 0.52 to 0.78, with a mean of 0.68 (Fig. 1, left). The mean of self-relationships was not equal to zero, which was expected because G was scaled using the sum of expected SNP variance assuming HWE ($\sum (2p_{i}\left(1-p_{i}\right)$), while the sum of true SNP variance is smaller in crossbreds because they deviate from HWE. The off-diagonal elements of G (i.e. pairwise relationships between individuals) ranged from −0.13 to 0.46, with a mean of 0.00 (Fig. 1, right). In the off-diagonals, 4.5 % of the pairwise relationships were equal to or higher than 0.1, and about 0.2 % of the relationships were equal to or higher than 0.3, suggesting that there were some half-sib pairs in the data, but no to very few full-sib pairs, as would be expected.

**Fig. 2:**
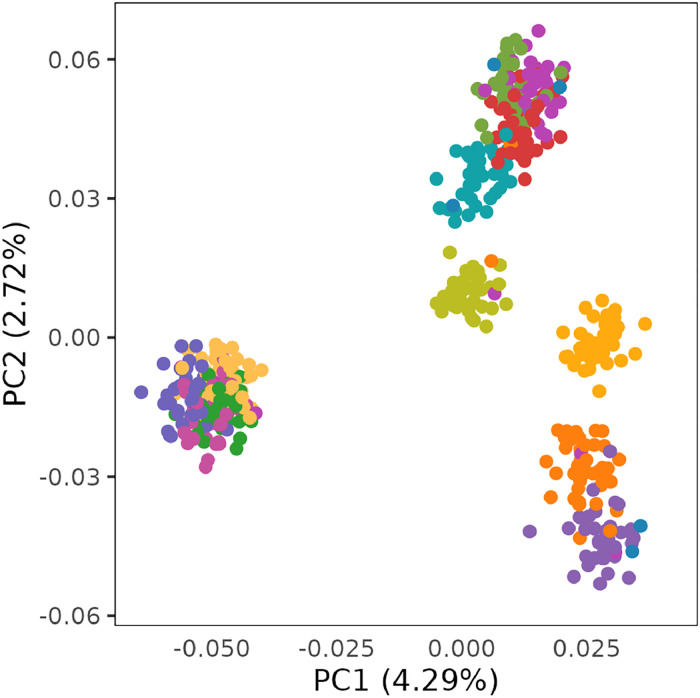
Principle component analysis of genomic relationships. First (x-axis) versus second (y-axis) principle components of the genomic relationships. Each dot represents an individual hen, and colors indicate their sires. The axis titles include the amount of variation explained by the two principal components.

The PCA on G revealed that the individuals clustered according to their sire half-sib relationships (Fig. 2). It is likely that some pairs of sires were related, because the offspring of certain sire pairs clustered more closely than others. The first and second principle components only explained 4.3 % and 2.7 % of the variation in relationships, respectively, suggesting that all individuals belonged to the same population.

### Descriptive statistics and estimated variance components

**Fig. 3:**
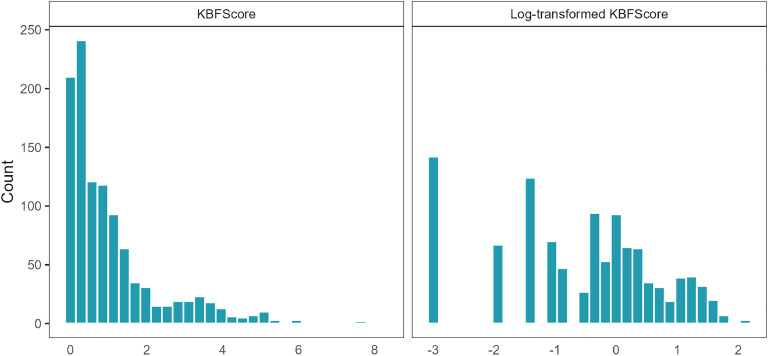
Trait distributions. Distribution of KBF Score (left), and the log-transformed KBF Score (right).

The original KBFScore values ranged from 0 to 8.2, with a mean of 1.06 and a standard deviation of 1.25 (Table 1 and Fig. 3, left). The log-transformed values (*logKBFScore*) ranged from −3 to 2.11, with a mean of −0.577 and standard deviation of 1.3 (Table 1 and Fig. 3, right).

**Table 1:** Descriptive statistics and estimated variance components KBFScore and logKBFScore.

Statistic	KBFScore		logKBFScore	
Minimum	0		−3	
Maximum	8.2		2.11	
Mean	1.06		−0.577	
Standard deviation	1.25		1.3	

Variance components	KBFScore		logKBFScore	
	Estimate	Standard error	Estimate	Standard error
Additive genetic variance	0.12	0.07	0.39	0.12
Residual variance	1.45	0.08	1.41	0.08
Heritability	0.08	0.04	0.22	0.06

The estimated heritability of *KBFScore* was lower than that of log*KBFScore* (0.08 versus 0.22), as well as the standard error of this estimate (0.04 versus 0.06)(Table 1). For *KBFScore,* the variance captured by the additive genetic effect ($\sigma_{u}^{2}$) was 0.12, which corresponds to an additive genetic standard deviation of 0.35. For log*KBFScore,* the variance captured by the additive genetic effect was equal to 0.39, which corresponds to an additive genetic standard deviation of 0.62.

### Genome-wide association study

**Fig. 4:**
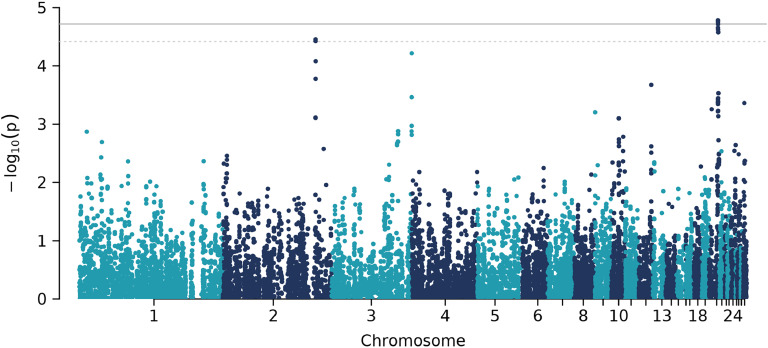
Manhattan plot for additive genetic effects.

**Fig. 5:**
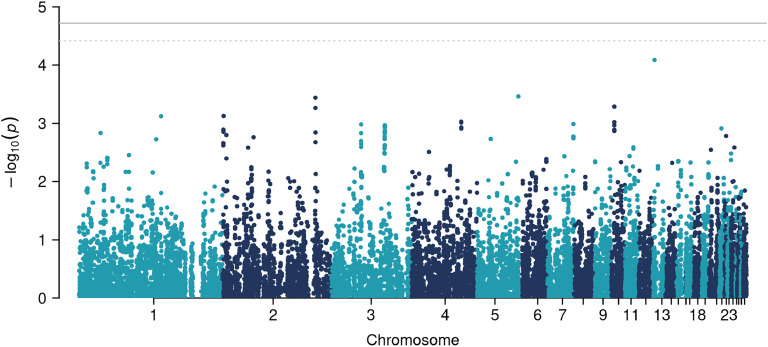
Manhattan plot for dominance genetic effects.

For additive genetic effects, the GWAS revealed that 9 SNPs on chromosome 20 were significantly associated with KBF (Table 2, Fig. 4, Fig. 5), and 2 SNPs on chromosome 2 were suggestively associated. None of the SNPs showed significant dominance genetic effects (table not shown). Although the original $-\log_{10}\left(p\right)$ values were inflated (β=1.27 for additive effects and β=1.1 for dominance effects), our empirical correction procedure effectively removed this inflation (Supplementary File 2: Fig. S1 and Fig. S2).

**Table 2:** GWAS table for additive genetic effects, showing SNPs that were significantly or suggestively associated with KBF.

Peak	Chr	Position	SNP	β	$-\log_{10}\left(p\right)$	$n_{0}$	$n_{1}$	$n_{2}$	$n_{\text{NA}}$	MAF
20_1	20	12324609	HGC_rs14280067	−0.2925	4.78	337	526	197	0	0.43
20_1	20	12333123	HGC039986	−0.2923	4.78	337	525	197	1	0.43
20_1	20	12349135	Gga_rs14280134	−0.2865	4.75	324	523	213	0	0.45
20_2	20	12423697	HGC_rs16174839	−0.2856	4.72	325	518	213	4	0.45
20_1	20	12299774	HGC039983	−0.2889	4.65	337	460	196	67	0.43
20_3	20	12458889	HGC040001	−0.2819	4.62	326	521	213	0	0.45
20_3	20	12695690	Gga_rs16175226	−0.2889	4.58	353	522	185	0	0.42
20_3	20	12697580	HGC_rs317841519	−0.2889	4.58	353	522	185	0	0.42
20_3	20	12762403	Gga_rs16175340	−0.2889	4.58	353	522	185	0	0.42
2_4	2	126429087	GGaluGA167797	−0.5875	4.45	638	392	30	0	0.21
2_4	2	126410011	HGC012247	−0.5862	4.42	609	388	30	33	0.22

Chr = chromosome. β = estimated regression coefficient. $n_{0},n_{1},n_{2}$ = number of hens with genotype 0, 1, and 2. $n_{\text{NA}}$ = number of hens with a missing genotype. MAF = minor allele frequency.

### Significant haplotype blocks and overlapping genes

Based on the location of SNPs that had a significant or suggestive association to KBF, we defined 4 distinct haplotype blocks on 2 chromosomes (Table 3). Together, the 68 SNPs in these haplotype blocks explained 13.2 % of the total additive genetic variance.

**Table 3:** Overview of the haplotype blocks that were found to harbor SNPs significantly associated with KBF, and the genes overlapping with these blocks (± 50 kb).

block	CHR	Start	Stop	Gene ENSID	strand	Gene Symbol
20_1	20	12269383	12367466	ENSGALG00000007786	-	DOK5
				ENSGALG00000042279	+	CYP24A1
				ENSGALG00000007796	+	BCAS1
				ENSGALG00000007792	-	PFDN4
20_2	20	12392696	12440981	ENSGALG00000054528	-	-
				ENSGALG00000042279	+	CYP24A1
				ENSGALG00000007796	+	BCAS1
20_3	20	12458889	12790234	ENSGALG00000054528	-	-
				ENSGALG00000030165	-	-
				ENSGALG00000040546	+	-
				ENSGALG00000032352	-	-
				ENSGALG00000025318	-	gga-mir0
				ENSGALG00000054108	-	-
				ENSGALG00000007796	+	BCAS1
				ENSGALG00000040886	+	-
				ENSGALG00000048250	+	-
				ENSGALG00000051448	+	-
				ENSGALG00000007809	-	TSHZ2
2_4	2	126410011	126634251	ENSGALG00000038971	-	CFAP418
				ENSGALG00000035901	-	GDF6
				ENSGALG00000040248	+	PLEKHF2

**Fig. 6:**
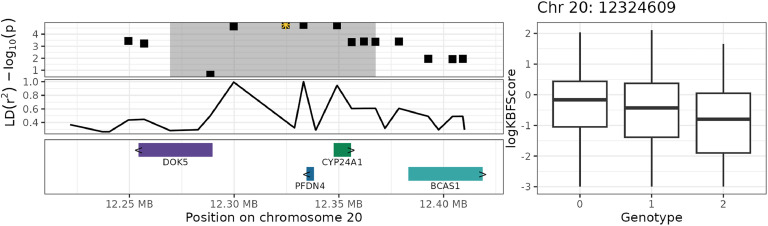
Genome plot for haplotype block 20_1 (grey area) on chromosome 20. The top-left plot shows the GWAS significance level in $-\log_{10}\left(p\right)$, where the yellow star indicates the top SNP with the strongest significance and the highlighted areas shows the haplotype block. The middle-left plot shows the strength of LD of SNPs with the top SNP. The bottom-left plot shows the genes and their names, with the arrows (> and <) indicating the direction of transcription. The right plot shows the raw logKBFScore values (y-axis) versus the genotype of the top SNP (x-axis).

**Fig. 7:**
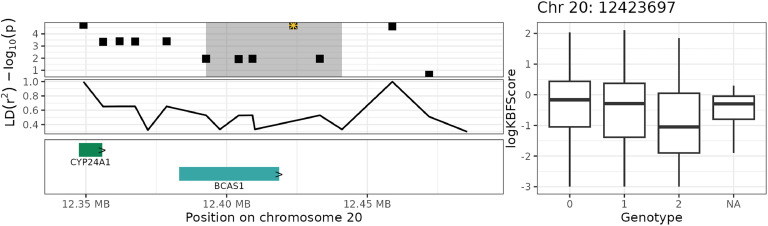
Genome plot for haplotype block 20_3 (grey area) on chromosome 20. The top-left plot shows the GWAS significance level in $-\log_{10}\left(p\right)$, where the yellow star indicates the top SNP with the strongest significance and the highlighted areas shows the haplotype block. The middle-left plot shows the strength of LD of SNPs with the top SNP. The bottom-left plot shows the genes and their names, with the arrows (> and <) indicating the direction of transcription. The right plot shows the raw logKBFScore values (y-axis) versus the genotype of the top SNP (x-axis).

**Fig. 8:**
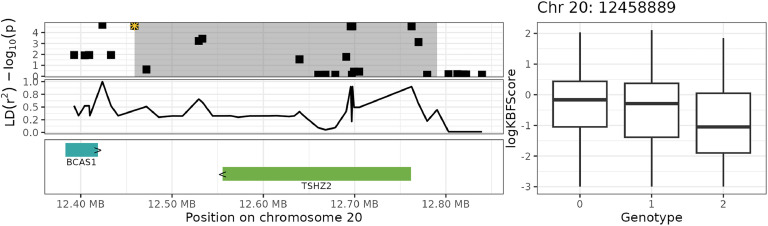
Genome plot for haplotype block 20_2 (grey area) on chromosome 20. The top-left plot shows the GWAS significance level in $-\log_{10}\left(p\right)$, where the yellow star indicates the top SNP with the strongest significance and the highlighted areas shows the haplotype block. The middle-left plot shows the strength of LD of SNPs with the top SNP. The bottom-left plot shows the genes and their names, with the arrows (> and <) indicating the direction of transcription. The right plot shows the raw logKBFScore values (y-axis) versus the genotype of the top SNP (x-axis).

**Fig. 9:**
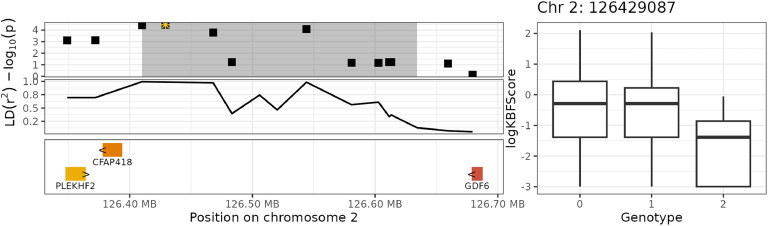
Genome plot for haplotype block 2_4 (grey area) on chromosome 2. The top-left plot shows the GWAS significance level in $-\log_{10}\left(p\right)$, where the yellow star indicates the top SNP with the strongest significance and the highlighted areas shows the haplotype block. The middle-left plot shows the strength of LD of SNPs with the top SNP. The bottom-left plot shows the genes and their names, with the arrows (> and <) indicating the direction of transcription. The right plot shows the raw logKBFScore values (y-axis) versus the genotype of the top SNP (x-axis).

The associated haplotype blocks overlapped or were in close proximity (± 50 kb) to 17 unique genes, of which eight had a known gene symbol. The three blocks on chromosome 20 overlapped with the genes DOK5, CYP24A1, BCAS1, PFDN4, and TSHZ2 (Figs. 6 and 7), and the block on chromosome 2 overlapped with the genes CFAP418, GDF6, and PLEKHF2 (Figs. 8 and 9). In the human GWAS catalog (Cerezo et al., 2024), the most frequently reported traits associated with these genes were glomerular filtration rate (13 studies), calcium measurement (11 studies), vitamin D measurement (10 studies), and serum creatinine amount (nine studies). Other relevant traits were body height (eight studies), body mass index (eight studies), BMI-adjusted waist-hip ratio (seven studies), and traits related to bone density (three studies). The most frequently reported associations involved the regions in or close to BCAS1, CYP24A1 and PFDN4, which are (like in chickens) located close to each other in the human genome (chromosome 20: 53943541–5421996). The intergenic and intragenic regions of these three genes have been associated with glomerular filtration rate (Loeb et al., 2024; Verma et al., 2024; Wuttke et al., 2019), calcium measurement (Sinnott-Armstrong et al., 2021; Verma et al., 2024), vitamin D measurement (Revez et al., 2020; Sinnott-Armstrong et al., 2021), and serum creatinine levels (Sinnott-Armstrong et al., 2021; Verma et al., 2024). The second most frequently reported gene was TSHZ2, which has been linked to BMI-adjusted waist-hip ratio (Christakoudi et al., 2021) and bone density in humans (He et al., 2023). The genes overlapping with the significant haplotype block on chromosome 2 also showed some interesting trait associations. The intergenic region between CFAP418 and PLEKHF2 has been associated with bone mineral accretion levels in humans (Park et al., 2015), and the intergenic region of CFAP418 has been associated with heel bone mineral density (Kim, 2018; Morris et al., 2019).

The MGI database reported 84 associations that involved the genes overlapping with haplotype blocks identified in the current study (The Jackson Laboratory, 2025). None of the reported traits were associated with more than two genes, so we decided to group traits based on their ontology to identify the most commonly reported trait groups (Supplementary File 1: Table S1). A large portion of associated traits related to skeletal development and bone morphology (involving GDF6 and CYP24A1), including abnormalities in ossification, joint and digit formation, and malformations of specific bones like carpal, tarsal, and middle ear ossicles. These associated traits suggest a strong link between the identified genes and pathways involved in bone growth and structural integrity. Another major group involves sensory function (involving GDF6, CFAP418 and PLEKHF2), especially related to vision and hearing, while additional clusters of traits point to metabolic processes, such as abnormal vitamin D levels and altered calcium metabolism (involving CYP24A1). Finally, several traits indicate early lethality or impaired development (involving GDF, CYP24A1, and TSHZ2), with reports of preweaning or postnatal lethality, embryonic failure, and growth retardation.

The haplotype blocks were aligned with QTL reported in the ChickenQTLdb, which revealed that the three significant blocks on chromosome 20 overlapped with QTL associated with age at first egg (Chen et al., 2024), skin color (Cha et al., 2023), bursa of fabricius weight (Sun et al., 2016), and egg number (Dong et al., 2019).

## Discussion

The objective of this study was to estimate the heritability of KBF susceptibility in commercial white-feathered laying hens, and to identify genomic regions and candidate genes associated with KBF. We found that KBF was low to moderately heritable, with an estimated heritability of 0.08 on the original traits scale, and 0.22 on the log-transformed scale. These results indicate a significant genetic component to KBF, which is consistent with other studies indicating genetic variation for bone strength and keel bone damage (Candelotto et al., 2017; Stratmann et al., 2016). Our genome-wide association study (GWAS) identified four genomic regions located on chromosomes 2 and 20 that were associated with KBF. The SNPs in these regions together explained 13.2 % of the additive genetic variance. The associated regions contained several genes (including BCAS1, CYP24A1, PFND4, TSHZ2, and GDF6) that have been linked to calcium and vitamin D homeostasis, skeletal development, and bone density in humans and mice. Furthermore, one of the genomic regions on chromosome 20 was in close proximity to QTL that have previously been associated with egg production levels and age at sexual maturity in chickens.

To our knowledge, the present study is the first to report heritability for KBF susceptibility in laying hens, which aligns with previously reported heritabilities for bone strength and bone density traits in chickens, ranging from 0.13 to 0.45 (Johnsson, 2020). Based on the estimated additive genetic standard deviation, the trait expressed on the original scale (*KBFScore)* had a genetic coefficient of variation of ∼33 %, supporting the feasibility of selection for reduced KBF susceptibility within commercial breeding programs. However, practical selection will require reliable and scalable phenotyping methods. While radiographic assessment of KBF is reliable (Rufener et al., 2018), it may not be feasible for high-throughput commercial application. For commercial application, data on KBF occurrence needs to be routinely measured in a commercial aviary setting, which is difficult and costly to realize. Furthermore, pedigree or genotype data is typically not collected from commercial crossbreds, which makes it difficult (if not impossible) to use crossbred phenotypes for genetic selection in the purebred nucleus population. Future research should prioritize the development of practical, objective, and high-throughput methods to assess keel bone status. Such a method could then be used to measure KBF at recurrent test farms. Alternatively, selection for reduced KBF susceptibility could be realized through identifying indicator traits that are genetically correlated with bone strength or KBF, such as easy-to-measure skeletal properties (Maidin et al., 2025), eggshell quality (Candelotto et al., 2017) or behavioral proxies such as altered movement patterns (Montalcini et al., 2024). For all these scenarios, genomic selection is probably the most promising approach for cost-effective selection for decreasing KBF susceptibility.

Some of the identified genes have been directly associated with bone related traits in mouse and humans. For example, studies in mouse models have associated *GDF6* (chromosome 2) and *CYP24A1* (chromosome 20) with skeletal development and bone shape (Baldarelli et al., 2024; The Jackson Laboratory, 2025). Furthermore, the gene *TSHZ2* (chromosome 20) has been linked to bone density in humans (He et al., 2023), suggesting that bone density is an important factor determining KBF susceptibility. Indeed, bone mineral density has been significantly correlated to bone breaking strength in laying hens (Jansen et al., 2020a). In addition to direct associations with bone traits, some of the identified genes may play a role in metabolic pathways that indirectly affect bone quality. For example, human GWAS studies have linked regions near *CYP24A1* and *BCAS1* to calcium balance and vitamin D levels (Sinnott-Armstrong et al., 2021; Verma et al., 2024). Vitamin D is essential for calcium absorption and bone mineralization (Bikle, 2012), so genetic mutations resulting in problems in this pathway could lead to weaker bones. In poultry, this metabolic stress is likely most acute during periods of peak lay, before full keel ossification. Our findings support the hypothesis that susceptibility to KBF is determined not only by genes influencing bone strength directly, but also by genes affecting the vitamin D metabolic pathway that regulates calcium reabsorption during lay.

The QTL for KBF we identified on chromosome 20 is located near a previously reported QTL for age at first egg (Chen et al., 2024) and egg number (Dong et al., 2019) in chickens. These results are in line with other studies that suggested that susceptibility to KBF in laying hens is indirectly caused by high levels of sustained egg production (Eusemann et al., 2020; Tixier-Boichard, 2020). In contrast, Jansen et al. (2020a) found no effect of total eggshell production on bone breaking strength or bone mineral density across different chicken lines, indicating that high egg yield does not necessarily compromise bone health. Furthermore, although Dunn et al. (2021) reported no significant genetic or phenotypic relationships between post-peak egg production and bone quality in White Leghorn and Rhode Island Red breeds, they did observe a significant negative genetic correlation between pre-peak production and bone quality in White Leghorns. Taken together, our findings and those of earlier studies suggest that while high and sustained egg production persistency may not directly impact bone quality, factors such as the timing of sexual maturity and the onset of lay may be important determinants of KBF risk.

In this study, we observed a relatively low incidence of KBF, as shown by the skewed distribution of KBF scores and the high peak at a KBF score of zero. The incidence was lower than some other reports in literature (Rufener and Makagon, 2020; Thøfner et al., 2021), which could be explained by the timing of KBF assessment, as KBF prevalence increases with hen age (Baur et al., 2020; Rufener and Makagon, 2020). In this study, KBF was measured at 30 weeks of age, which coincides with a period where prevalence is typically still increasing.

Our GWAS results showed high genomic inflation factors (β=1.27 for additive effects and β=1.1 for dominance effects) before we applied an empirical correction. Such inflation can be caused by population stratification (or more general, variation in relationships across pairs of individuals) that is not fully captured by the model (Hellwege et al., 2017). One strategy to correct for relatedness is to include a random effect with a genomic relationship matrix as variance structure. In this study, however, population stratification was limited, as evidenced by the first two principal components of the GRM that only explained 7 % of the variation. We therefore decided to not correct for population stratification, but to apply an empirical post-hoc correction of p-values. Alternatively, high genomic inflation factors could be a consequence of strong linkage disequilibrium (LD) in the crossbred genotypes. A study in mice found that hybrids such as F2 populations are more susceptible to high genomic inflation when compared to outbred populations (Tyler et al., 2021). Furthermore, KBF is likely a complex trait influenced by many genes. For such traits, a slight inflation of test statistics across the genome can sometimes be expected (Yang et al., 2011).

Keel bone fractures in laying hens are closely linked to behaviors that can both contribute to and result from these injuries. Hens with KBF often exhibit increased fearfulness and anxiety, as evidenced by longer durations in tonic immobility tests and heightened latency to approach novel objects, indicating elevated stress levels (Nasr et al., 2012; Wei et al., 2022). These behavioral alterations may stem from the pain associated with fractures, leading to decreased vertical movements (Montalcini et al., 2024) and more time spent in the top tier of the barn (Rufener et al., 2019). Conversely, Baker et al. (2024) demonstrated that behaviors such as collisions with housing structures and aggressive interactions significantly increase the likelihood of hens developing KBF. Their study found that the number of collisions and aggressive encounters a hen experiences are predictive of fracture occurrence, independent of the impact's magnitude. This finding aligns with our own, which identified genes related to vision and hearing within the KBF-associated genomic regions. It is plausible that impaired vision or hearing in some hens could increase the frequency of collisions and aggressive encounters, thus raising their susceptibility to fractures. In contrast, Montalcini et al. (2024) found no evidence that spatial behaviors such as vertical locomotion (jumping and flying) are associated with fracture severity. Finally, hens with bumblefoot are more likely to develop KBFs, possibly due to altered perching behaviors that increase the risk of falls or collisions (Gebhardt-Henrich and Fröhlich, 2015). The contrasting findings of these studies underscore the complex interplay between behavior and KBFs, highlighting the need for management practices that consider both behavioral and physiological factors to mitigate fracture risk and enhance hen welfare. Future genetic studies could focus on estimating genetic correlations of KBF with production and behavioral traits, and the possible link with impaired vision and hearing to improve our understanding of the etiology of KBF.

In conclusion, we found that KBF is low to moderately heritable in laying hens, confirming that genetic factors play an important role in fracture susceptibility. The substantial genetic variation for this trait supports the feasibility of selection for reduced KBF susceptibility within commercial breeding programs. Our genome-wide association study identified four genomic regions on chromosomes 2 and 20 that explained 13.2 % of genetic variance, and contained several candidate genes involved in calcium metabolism, vitamin D pathways, and bone development in other species. In addition, one of the genomic regions on chromosome 20 was in close proximity to QTL that have previously been associated with egg production levels and age at sexual maturity in chickens. Together, our findings contribute to our understanding of the genetic architecture of KBF susceptibility, its possible relationship to early egg production, and the physiological pathways that influence skeletal health in laying hens. Future research should develop practical methods to assess KBF in large commercial flocks with high reliability. Furthermore, the candidate genes we identified need further study to understand their specific functions in chicken bone metabolism. Finally, future studies should also examine other traits that correlate with KBF, such as bone or eggshell quality, as alternative selection criteria.

## Declaration of AI and AI-assisted technologies in the writing process

During the preparation of this work the authors used Gemini Flash 2.5 in order to receive feedback on written text, and to provide suggestions for improving the written text. In addition, the authors used Claude Sonnet 3.7 to group traits from the MGI database into trait categories. After using these tools, the authors reviewed and edited the content as needed and take full responsibility for the content of the publication.

## CRediT authorship contribution statement

**Pascal Duenk:** Writing – original draft, Visualization, Methodology, Investigation, Formal analysis, Data curation, Conceptualization. **Henk Bovenhuis:** Writing – review & editing, Supervision. **Pauline Willemsen:** Writing – review & editing, Resources, Data curation. **Bayode O. Makanjuola:** Writing – review & editing, Data curation. **Matthew B. Petelle:** Writing – review & editing, Resources, Data curation. **Sabine G. Gebhardt-Henrich:** Writing – review & editing, Resources, Data curation. **Christine F. Baes:** Writing – review & editing, Supervision. **Michael J. Toscano:** Writing – review & editing, Resources, Data curation.

## Disclosures

The authors declare that they have no known competing financial interests or personal relationships that could have appeared to influence the work reported in this paper.

## Appendix Supplementary materials

Supplementary File 1: QQ-plots. Image, application 1

Supplementary File 2: trait categories MGI Image, application 2
