# Supplementary material for: Genetic analysis of keel bone fractures in laying hens housed in a quasi-commercial aviary
Source: Poult Sci. 2025 Nov 7;104(12):106067. doi: 10.1016/j.psj.2025.106067 (PMC12664023; doi:10.1016/j.psj.2025.106067)
Supplement: Supplementary file 1 — QQ-plots. [file mmc1.docx]

Table S 1 List of traits identified in the MGI database, grouped into trait categories

| **Trait Category** | **Associated Traits** |
| --- | --- |
| Skeletal Development & Bone Morphology | abnormal articular cartilage morphology, abnormal bone ossification, abnormal carpal bone morphology, abnormal digit morphology, abnormal incudomalleolar joint morphology, abnormal incudostapedial joint morphology, abnormal incus morphology, abnormal malleus morphology, abnormal middle ear ossicle morphology, abnormal skeleton morphology, abnormal stapes annular ligament morphology, abnormal stapes morphology, abnormal tarsal bone morphology, absent coronal suture, absent stapes annular ligament, fused carpal bones, fused joints, fused tarsal bones, small incus, small malleus, small middle ear ossicles, small stapes |
| Sensory Function: Vision | abnormal cone electrophysiology, abnormal electroretinogram waveform feature, abnormal optic disk morphology, abnormal photoreceptor outer segment morphology, abnormal retina blood vessel morphology, abnormal retina morphology, abnormal retina photoreceptor morphology, abnormal rod electrophysiology, decreased a-wave amplitude, decreased b-wave amplitude, decreased retina photoreceptor cell number, decreased total retina thickness, retina degeneration, short photoreceptor outer segment, thin retina outer nuclear layer |
| Sensory Function: Hearing | abnormal auditory brainstem response, abnormal hearing physiology, small incus, small malleus, small stapes, abnormal incus morphology, abnormal malleus morphology, abnormal stapes morphology, abnormal middle ear ossicle morphology, abnormal incudomalleolar joint morphology, abnormal incudostapedial joint morphology, absent stapes annular ligament |
| Metabolic & Nutritional Traits | abnormal vitamin D level, increased circulating alkaline phosphatase level, increased circulating calcium level, increased urine calcium level, decreased circulating HDL cholesterol level, decreased circulating cholesterol level, increased circulating unsaturated transferrin level |
| Immune & Organ Morphology | abnormal kidney morphology, abnormal lung morphology, abnormal spleen morphology, abnormal thymus morphology, enlarged spleen, enlarged thymus, small kidney, dilated renal tubule |
| Neural Development & Function | abnormal axon morphology, abnormal brain morphology, brain inflammation, decreased myelin sheath thickness, dysmyelination, hydrocephaly, decreased prepulse inhibition, decreased startle reflex, decreased anxiety-related response, decreased exploration in new environment |
| Development & Survival | embryonic lethality between implantation and somite formation (complete penetrance), failure of blastocyst to hatch from the zona pellucida, postnatal growth retardation, postnatal lethality, postnatal lethality (incomplete penetrance), preweaning lethality (complete penetrance), preweaning lethality (incomplete penetrance), decreased body size, decreased body weight, no spontaneous movement |
| Hematological Traits | decreased mean corpuscular hemoglobin, decreased mean corpuscular volume, increased red blood cell distribution width |
