# Supplementary material for: Genetic analysis of keel bone fractures in laying hens housed in a quasi-commercial aviary
Source: Poult Sci. 2025 Nov 7;104(12):106067. doi: 10.1016/j.psj.2025.106067 (PMC12664023; doi:10.1016/j.psj.2025.106067)
Supplement: Supplementary file 2 — trait categories MGI [file mmc2.docx]

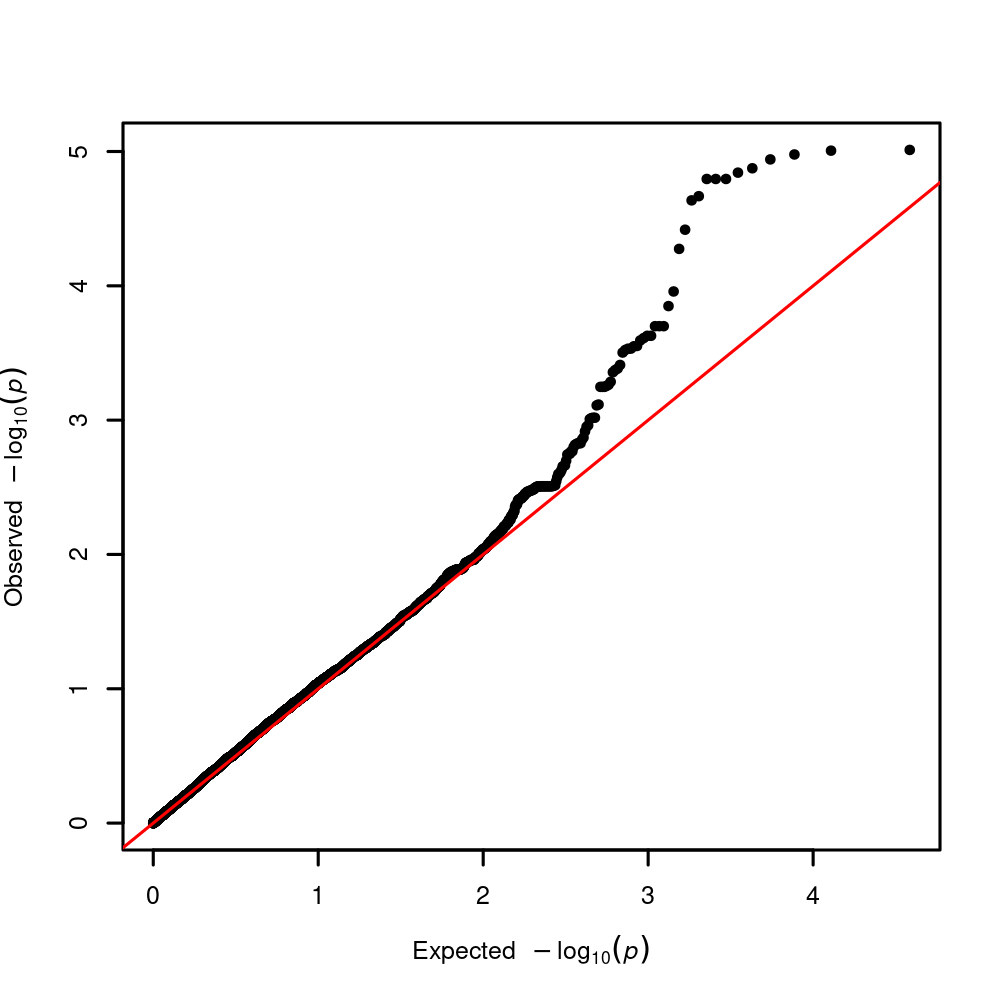


Figure S 1 qqplot for additive genetic effects after correcting for genomic inflation.


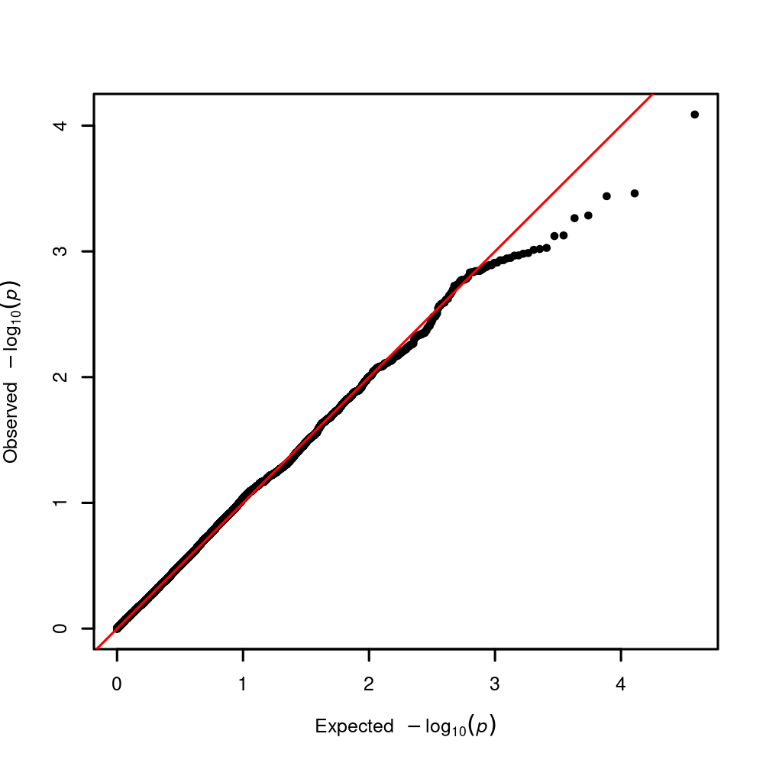


Figure S 2 qqplot for dominance genetic effects after correcting for genomic inflation.
